# PTTG1 cooperated with GLI1 leads to epithelial-mesenchymal transition in esophageal squamous cell cancer

**DOI:** 10.18632/oncotarget.21343

**Published:** 2017-09-27

**Authors:** Wang Feng, Xuan Xiaoyan, Li Shenglei, Liu Hongtao, Jiang Guozhong

**Affiliations:** ^1^ Department of Oncology, First Affiliated Hospital of Zhengzhou University, Zhengzhou 450052, P.R. China; ^2^ Department of Immunology, School of Basic Medical Science, Zhengzhou University, Zhengzhou 450001, P.R. China; ^3^ Department of Pathology, The First Affiliated Hospital of Zhengzhou University, Zhengzhou 450052, P.R. China; ^4^ Laboratory for Cell Biology, School of Life Sciences of Zhengzhou University, Zhengzhou 450001, P.R. China

**Keywords:** esophageal squamous cell cancer, pituitary tumor-transforming gene-1, glioma-associated oncogene homolog1, epithelial-mesenchymal transition

## Abstract

Pituitary tumor-transforming gene-1 (PTTG1) could acquire its metastasis-promoting effects via inducing epithelial-mesenchymal transition (EMT). However, its role and mechanism in EMT in esophageal squamous cell cancer (ESCC) had not been clearly elucidated. Here, we demonstrated that PTTG1 was overexpressed in ESCC cell lines and tissues especially those with lymph node metastasis. Down regulation of PTTG1 levels dampened the ESCC cells invasion, migration, proliferation ability and colony formation *in vitro* and inhibited the growth of mouse xenograft model of ESCC cells *in vivo*. In addition, our *in vitro* and *in vivo* experiments consistently showed that decreased PTTG1 led to the inhibition of EMT process. Glioma-associated oncogene homolog1 (GLI1), a key factor in HH-GLI signaling pathway, was also overexpressed in ESCC cells and tissues. Mechanistic studies demonstrated that decreased PTTG1 mitigated the expression levels of GLI1 *in vitro* and *in vivo* and ChIP assay also indicated that PTTG1 cooperated with GLI1 by binding to its promoter. Furthermore, overexpression of GLI1 rescued the EMT inhibited by down regulation of PTTG1 *in vitro*. Together, these data suggested that PTTG1 promoted the invasion ability of ESCC cells via EMT, more important, PTTG1 participated in EMT via activating the expression of GLI1 in ESCC. PTTG1 could be a candidate biomarker for defining ESCC metastasis and useful target for therapy.

## INTRODUCTION

Esophageal squamous cell cancer (ESCC) is one of the most malignant diseases in China, causing more than 400000 deaths per year [1–2]. Although great efforts have been made to improve the treatment of patients with ESCC, the 5-year-survival rate is also low for those with invasion and metastasis [3]. So it is urgent for us to achieve timelier and earlier-stage diagnosis.

Epithelial-mesenchymal transition (EMT), originally a normal cell differentiation process during early development, has been described in invasion and metastasis in certain cancers [4–6]. In cancer cells, EMT can be awoken and make the cancer cells lose their epithelial phenotype, obtain mesenchymal phenotype and acquire metastasis ability in the end [6]. Switch on EMT in cancer cells is induced by many different signaling pathways after these pathways are activated by various stimuli [7].

Pituitary tumor-transforming gene-1 (PTTG1) was firstly identified and characterized in rat pituitary tumor cells [8]. PTTG1 which expressed at very undetectable or low level in normal tissues, was shown to be highly expressed in a variety of cancers tissues: colorectal, ovarian, breast, colon and esophagus, what's more its expression was also associated with lymph node metastasis [9–12]. Yoon et al showed that PTTG1 expression played an important role in the metastasis of breast cancer cells at least in part through regulation of EMT [11]. Shah et al also demonstrated that PTTG1 was a key factor in the induction of EMT in colon cancer [12]. The role of PTTG1 in the invasion and metastasis of ESCC has been reported before [13, 14], however, the role and mechanism of PTTG1 in the occurrence of EMT in ESCC have not been elucidated to date.

During EMT process, a great deal of cancer signaling pathway, such as TGF-β, NOTCH, Wnt, PI3K-Akt all have crosstalk with Hedgehog (HH)/glioma-associated oncogene homolog (GLI) pathway and the interaction among these signaling pathway plays pivotal roles for the induction of EMT and tumor aggressiveness [15]. The HH-GLI1 signaling pathway, mostly quiescent in grownups, is always inappropriately deregulated in most cancers and plays an important role in the development and function of many organs: cell proliferation, apoptosis, survival and differentiation [16–18]. Three members of GLI family of transcription factors have been found to date, GLI1, GLI2 and GLI3, while GLI1 is the only one with full-length transcriptional activator. As GLI1 is not only a target gene of HH signaling but also a complete transcriptional activator, it is deemed as a measure of HH signaling activity [19–20]. Now, GLI1 has been found to be overexpressed in many kinds of cancers include ESCC. Furthermore, overexpression of GLI1 is often associated with the metastasis ability of cancers [21–22]. Lately, the HH-GLI1 signaling pathway has been found to play a significant role in the induction of EMT. Endothelial cells with GLI1 excessive activation could exhibit mesenchymal transformation in phenotype, morphology and function [23].

Recently, researchers have shown that PTTG1 could cooperate with some signaling pathway, such as: Rho/ROCK and RAS-MAPK to induce EMT and promote tumorigenesis in cancers. However, it is not known whether there has interaction between PTTG1 and HH-GLI1 signaling pathway in the occurrence of EMT and whether the interaction between PTTG1 and HH-GLI1 signaling pathway could promote invasion and metastasis in ESCC through EMT. Here, we demonstrated that PTTG1 and GLI1 were all overexpressed in ESCC tissues and cell lines. Down regulation of PTTG1 significantly decreased the activity of GLI1, occurrence of EMT and the growth of ESCC cells *in vitro* and *in vivo*. In addition, up-regulation of GLI1 partially rescued the EMT inhibited by PTTG1. Therefore, there existed cooperation between PTTG1 and GLI1 signaling pathway, up-regulation of PTTG1 could induce EMT and invasion and metastasis in ESCC through activation of GLI1signaling pathway. Both PTTG1 and GLI1 signaling pathway could be potential therapeutic strategy for ESCC.

## RESULTS

### The expression levels of PTTG1 and GLI1 in ESCC tissues and ESCC cell lines

**Figure 1:**
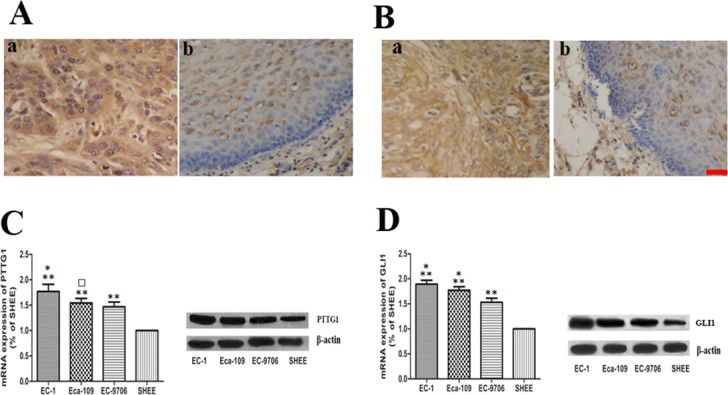
The expression of PTTG1 and GLI1 in ESCC tissues and cell lines **(A, B)** Localization of PTTG1 and GLI1 in benign and malignant esophageal tissues with immunohistochemical stains. **(Aa)** Predominantly the cytoplasmic staining of PTTG1 in ESCC tissues. (×400). **(Ab)** The staining of PTTG1 in benign esophageal epithelium (x400). **(Ba)** Predominantly the cytoplasmic staining of GLI1 in ESCC tissues. (×400). **(Bb)** The staining of GLI1 in benign esophageal epithelium (x400). **(C)** Real time RT-PCR and western blot all showed increased expression of PTTG1 in ESCC cell lines (^**^p<0.01). Furthermore, EC-1 with the highest invasion and metastasis ability showed the highest expression of PTTG1 (^*^p<0.05). The expression level of PTTG1 in EC-109 was higher than those in EC-9706, but the difference was not statistically significant (□p>0.05). **(D)** Real time RT-PCR and western blot also showed that the expression level of GLI1 in ESCC cell lines were higher than those in immortalized human esophageal epithelial cell line SHEE (^**^p<0.01). In addition, the expression of GLI1 in EC-1 was higher than those in EC-109 and the expression of GLI1 in EC-109 was higher than those in EC-9706 (^*^p<0.05). Bar=50μm.

The expression levels of PTTG1 and GLI1 in ESCC tissues and adjacent esophageal mucosa were determined by immunohistochemistry method. The expression levels of PTTG1, predominantly localized in the cytoplasm, were significantly up-regulated in ESCC tissues than those in adjacent esophageal mucosa (Figure 1A). Furthermore, the up-regulation of PTTG1 was positively correlated with lymph node metastasis (Table 1). We got the same results with GLI1, the expression levels of GLI1 in ESCC tissues were higher than those in adjacent esophageal mucosa (Figure 1B) and the higher expression of GLI1 was positively correlated lymph node metastasis (Table 1). Significantly, positive relationship was found between PTTG1 and GLI1 expression. In order to further explore the role of PTTG1 and GLI1 in the invasion and metastasis of ESCC, we detected the expression levels of PTTG1 and GLI1 in three ESCC cell lines with different metastasis ability and immortalized human esophageal epithelial cell line SHEE. The mRNA and protein expression levels of PTTG1 and GLI1 in EC-1, EC9706, Eca-109 and SHEE were shown in Figure 1C, 1D. EC-1 with the highest metastasis ability showed the highest PTTG1 and GLI1 mRNA and protein expression, while the PTTG1 and GLI1 mRNA and protein expression in SHEE showed no or lowest expression. So EC-1 and Eca-109 were chosen for the following experiments. The above results demonstrated that ESCC metastasis was associated with significantly increased expression of PTTG1 and GLI1.

**Table 1:** Frequencies of PTTG1 and GLI1 protein expression in benign and malignant esophageal tissues

	Cases ^#^	PTTG1	GLI1
Normal	50	17(34.0)	20(40.0)
ESCC	50	31(62.0)^*^	37(74.0)^*^
ESCC LN+	34	24(70.6) ^#^	28(82.4)
ESCC LN-	16	7(43.8)	9(56.3)

ESCC: esophageal squamous cell carcinoma; Normal: normal esophageal mucosa; LN+: with lymph node metastasis; LN-: without lymph node metastasis; ^*^ vs normal p<0.01, ^#^vs ESCC LN-, p<0.05.

### Down regulation of PTTG1 levels decreased ESCC cell invasion, migration and proliferation ability *in vitro*

**Figure 2:**
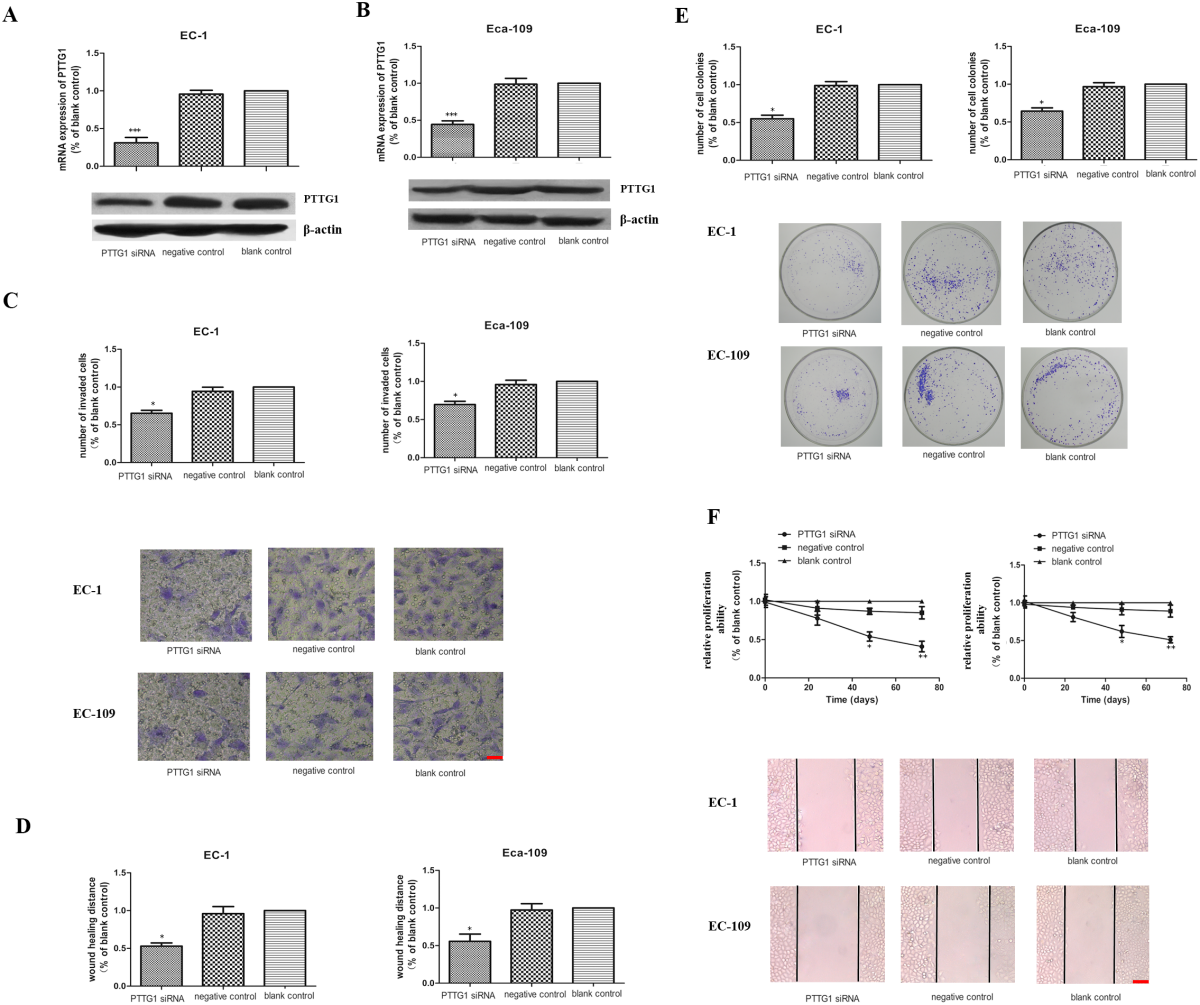
The role of PTTG1 in the invasion, migration and proliferation abilities in EC-1 and EC-109 cells *in vitro* **(A, B)** Real time RT-PCR and western blot results demonstrated that the PTTG1 mRNA and protein levels were significantly reduced in EC-1 and EC-109 cells transfected of PTTG1 siRNA (^***^p<0.001). **(C)** Cell invasion assay showed that the number of invaded cells in EC-1 and EC-109 cells transfected of PTTG1 siRNA were greatly fewer than those in control groups (^*^p<0.05). Representative pictures of cells invaded through the filter were shown here. Bar=20μm. **(D)** The results of the wound healing assay also showed the wound healing ability of EC-1 and EC-109 cells transfected of PTTG1 siRNA were weaker than those in control groups (^*^p<0.05). Representative pictures of cells migrated to the wounded area were shown here. Bar=100μm. **(E)** Colony formation assay revealed that the colonies formed in EC-1 and EC-109 cells transfected of PTTG1 siRNA were smaller and fewer than those in control groups (^*^p<0.05). Representative pictures of cell colonies formed in different groups were shown here. **(F)** CCK8 assay showed that the proliferation abilities of EC-1 and EC-109 cells transfected of PTTG1 siRNA were decreased compared to those in control groups, especially at 48h and 72h (^*^p<0.05).

To further explore the role of PTTG1 in the progression of ESCC, especially its function in the invasion and metastasis of ESCC, siRNA targeting PTTG1 was transiently transfected into EC-1 and Eca-109 cells for 48h. As shown in Figure 2A, 2B, the mRNA and protein expression levels of PTTG1 were significantly decreased in EC-1 and Eca-109 cells transfected with PTTG1 siRNA compared with those in control groups. Furthermore, with the down regulation of PTTG1, the invasion and metastasis ability of EC-1 and Eca-109 cells were also decreased greatly in comparison with those in control groups (Figure 2C, 2D). Cell metastasis is always closely related to their proliferation ability. So we tested the colony formation rate and colony size in ESCC cell lines. After transfected with PTTG1 siRNA, the number of colonies formed by EC-1 and Eca-109 were also significantly decreased (Figure 2E). In addition, the size of colonies of EC-1 and Eca-109 cells transfected with PTTG1 siRNA was clearly minified compared with those in negative and blank control. Proliferation ability of EC-1 and Eca-109 cells were also reduced with the lower expression of PTTG1 (Figure 2F). Thus the down regulation of PTTG1 could not only decrease the invasion and metastasis ability but also the proliferation ability of the ESCC cells.

### Down regulation of PTTG1 levels inhibited the induction of EMT *in vitro*

**Figure 3:**
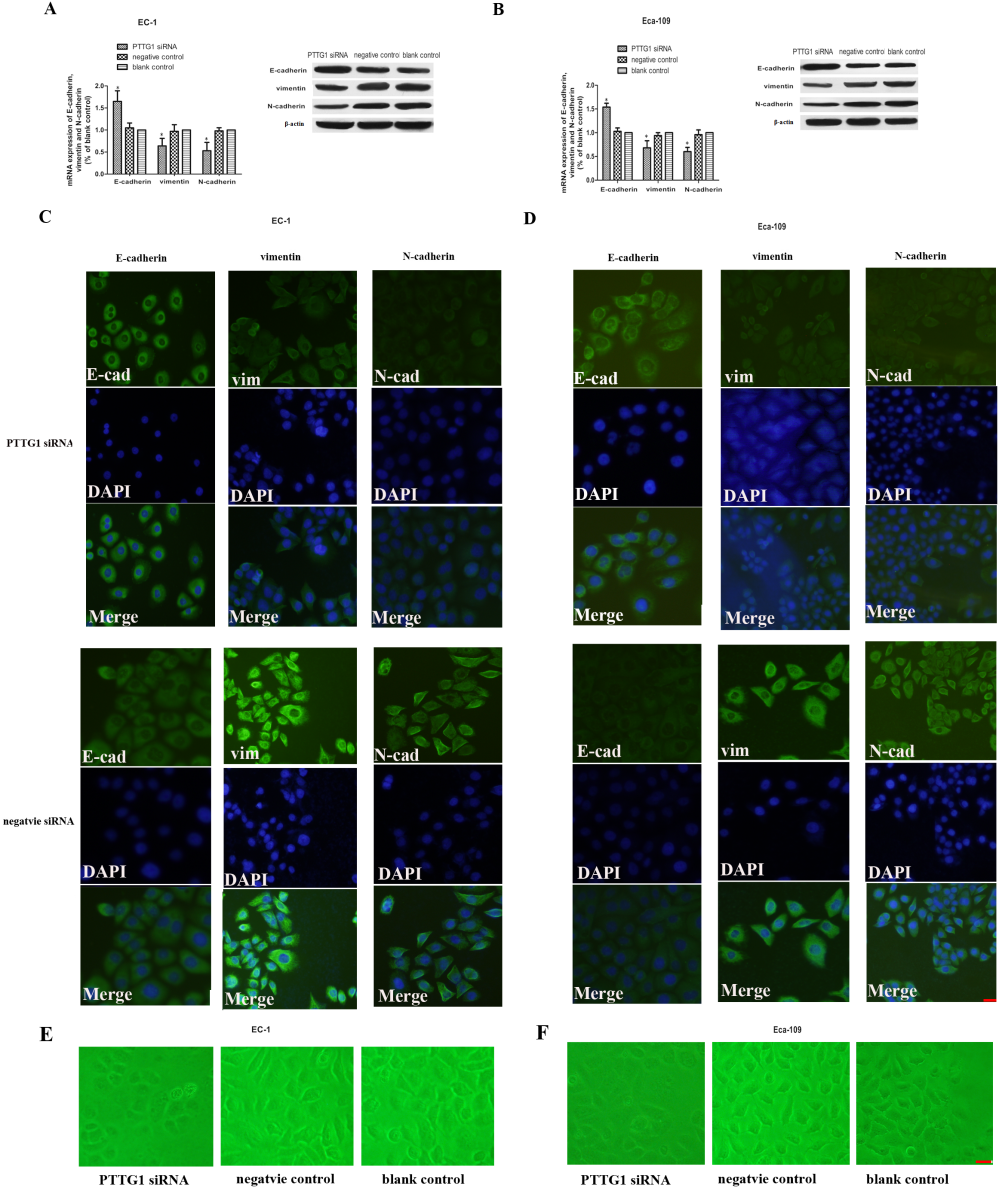
Down regulation of PTTG1 altered the expression of E-cadherin, vimentin and N-cadherin **(A, B)** Real time RT-PCR and western blot results demonstrated that with the down regulation of PTTG1, the mRNA and protein expression of E-cadherin were significantly up regulated while the mRNA and protein expression of vimentin and N-cadherin were down regulated compared with those in control groups (^*^p<0.05). **(C, D)**. EC-1 and EC-109 cells were stained for the epithelial markers and the mesenchymal marker vimentin, N-cadherin. Down regulation of PTTG1 induced a switch of epithelial marker and mesenchymal markers in EC-1 and EC-109 cells. Representative field of view in EC-1 and EC-109 transfected with siRNA group or EC-1 and EC-109 transfected with negative siRNA were shown here. Bar=10μm **(E, F)**. PTTG1 siRNA blocked the morphological changes of EC-1 and Eca-109 and let them to be more like normal epithelial cells. Representative field of view in EC-1 and EC-109 transfected with siRNA group, EC-1 and EC-109 transfected with negative siRNA or vacant cells were shown here. Bar=10μm.

EMT is one of the critical steps in tumor invasion and metastasis. The process of EMT is critically regulated and variable genetic alterations were involved in this process. We have shown that down regulation of PTTG1inhibited the invasion and metastasis of ESCC, but whether PTTG1 participated in ESCC invasion and metastasis via EMT had not been studied until now. Therefore we checked the epithelial marker: E-cadherin and mesenchymal marker: vimentin and N-cadherin in EC-1 and Eca-109 cells in different groups. Compared with those in control groups, the mRNA and protein expression of E-cadherin in EC-1 and Eca-109 cells transfected with PTTG1 siRNA increased significantly, while the mRNA and protein expression of vimentin and N-cadherin in EC-1 and Eca-109 cells transfected with PTTG1 siRNA dampened dramatically (Figure 3A, 3B). The results of Immunofluorescence demonstrated that the membrane staining of E-cahderin was up regulated in EC-1 and Eca-109 cells transfected with PTTG1 siRNA compared with those in control groups which the staining of E-cadherin was mainly concentrated in the cytoplasm. While the staining of vimentin and N-cadherin in EC-1 and Eca-109 cells transfected with PTTG1 siRNA were transferred from cell membrane to cytoplasm and the fluorescence intensity decreased obviously compared with those in control groups (Figure 3C, 3D). Additionally, morphological changes of the EC-1 and Eca-109 cells could be blocked by PTTG1 siRNA. So we came to the conclusion that PTTG1 played as a promoter of EMT in ESCC (Figure 3E).

### Down regulation of PTTG1 levels inhibited the expression of GLI1 *in vitro*

**Figure 4:**
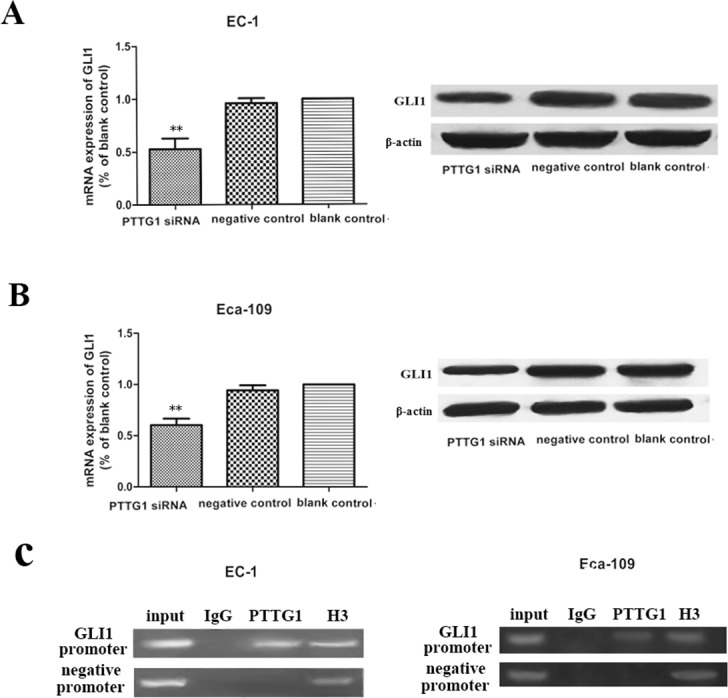
Down regulation of PTTG1 inhibited the mRNA and protein expression of GLI1 **(A, B)** Real time RT-PCR and western blot results demonstrated that with the down regulation of PTTG1, the mRNA and protein expression of GLI1were down regulated compared with those in control groups (^**^p<0.01). **(C)** Chromatin DNA in EC-1 and Eca-109 were immunoprecipitated by PTTG1 antibody and by H3 antibody. Enrichment of chromatins was got using primers targeting GLI1 promoter's PTTG1 binding site, but not with primers targeting other places on the promoters of GLI1.

Transcription program switching in EMT is always activated by many signaling pathways including transforming growth factor β (TGF-β), Wnt–β-catenin, Hedgehog, Notch and others. GLI1 is a key molecule which can translocate to the nucleus of cells and activates the transcription of HH-GLI1 signaling pathway target genes, thus the expression levels of GLI1 is often deemed as a measure for the HH signaling activity. Here, we found that with the decrease expression of PTTG1 in EC-1 and Eca-109 cells transfected with PTTG1 siRNA, the mRNA and protein expression of GLI1 was also reduced (Figure 4A, 4B). These data demonstrated that there existed interaction between PTTG1 and GLI1, and PTTG1 engaged in the induction of EMT in ESCC perhaps via the activation of HH-GLI1 signaling pathway.

Next, we used ChIP method to examine the link between PTTG1 and GLI1. We discovered that PTTG1 binds directly to the promoter of GLI1 in -841 to -665 region. Thus, our results reinforced our conclusion that PTTG1 cooperated with GLI1 via direct interaction with its promoter.

### Down regulation of PTTG1 levels decreased growth ability, EMT and activation of GLI1 *in vivo*

**Figure 5:**
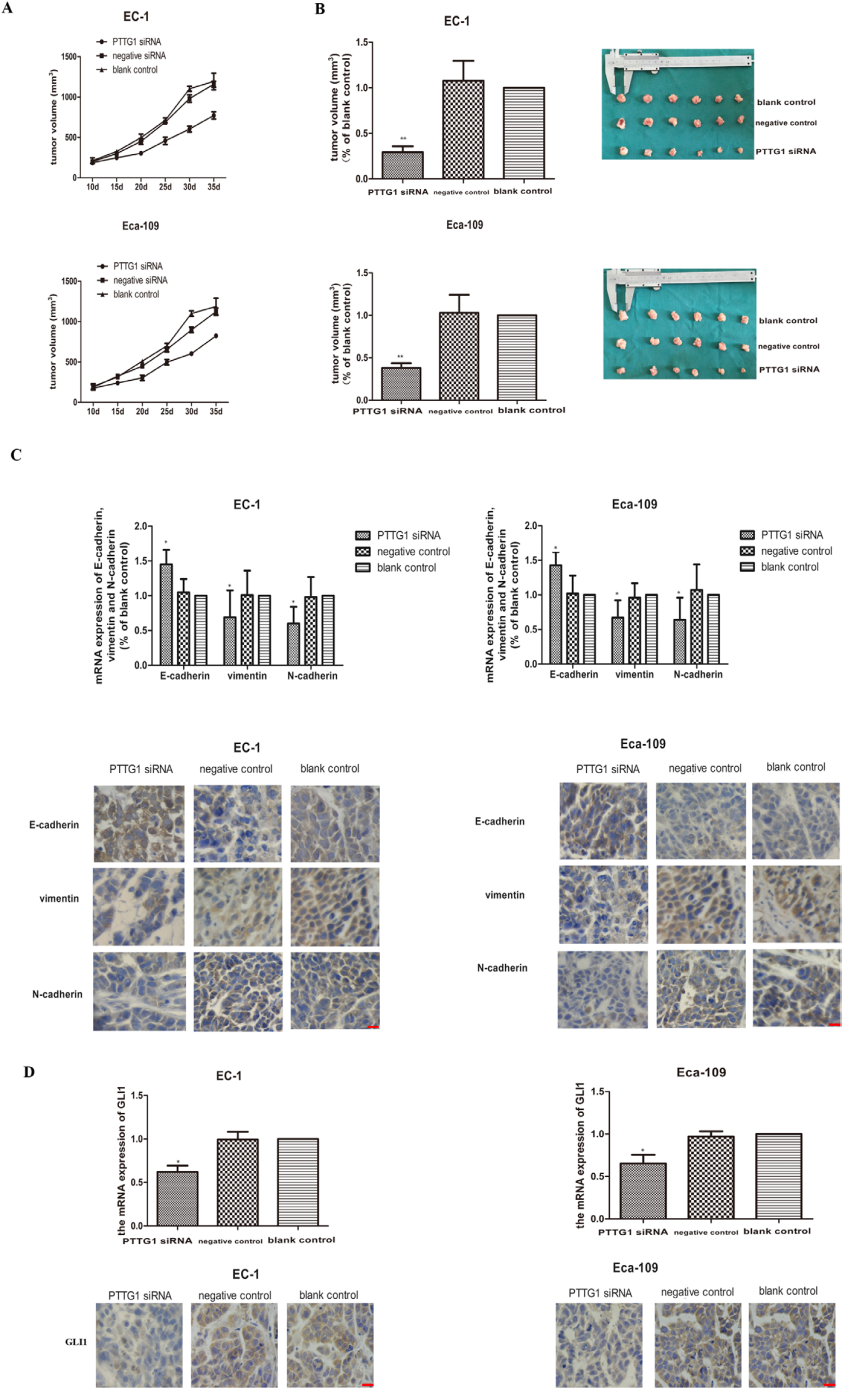
Down regulation inhibited the occurrence of EMT and xenograft growth *in vivo* **(A)** Tumor growth of EC-1 cells and EC-109 cells, tumor volumes were monitored every 5 days. **(B)** Xenograft were taken and weighed and the results showed that the weight of the xenograft of EC-1 and Eca-109 transfected with PTTG1 siRNA were apparently lighter than those in control groups (^**^p<0.01). **(C)** The mRNA and protein expression of E-cadherin, vimentin and N-cadherin were examined by real time RT-PCR and immunohistochemistry. And the results showed that PTTG1 also modulated the mRNA and protein expression of E-cadherin, vimentin and N-cadherin *in vivo*. With the down regulation of PTTG1, the mRNA and protein expression of E-cadherin were increased, while the mRNA and protein expression of were decreased greatly (^*^p<0.05). **(D)** The mRNA and protein expression of GLI1 were examined by real time RT-PCR and immunohistochemistry. And the results showed with the down regulation of PTTG1, the mRNA and protein expression of GLI1 were significantly decreased (^*^p<0.05). Bar=20μm.

The *in vivo* roles for PTTG1 were tested by injecting EC-1 and Eca-109 cells (transfected with PTTG1 siRNA, negative siRNA or vacant cells) subcutaneously into SCID mice (6 mice per group). Mice injected with PTTG1 siRNA transfected EC-1 or Eca-109 cells showed a significant delay in tumor development (Figure 5A). As shown in Figure 5B, the mean size of tumors in PTTG1 siRNA transfected EC-1 or Eca-109 cells’ groups were all about 70% smaller than the size of tumors in control groups. The levels of vimentin and N-cadherin were all reduced in PTTG1 siRNA transfected EC-1 or Eca-109 cells’ groups relative to the control tumors, while the most important marker of EMT, E-cadherin, expressed higher in PTTG1 siRNA transfected EC-1 or Eca-109 cells’ groups compared to those in control groups (Figure 5C), indicating decreased EMT in PTTG1 down-regulation tumors. To further understand the role of PTTG1 in the activation of HH-GLI1 signaling pathway, the mRNA and protein expression of GLI1 were detected. As expected, dampened GLI1 expression was observed in PTTG1 siRNA transfected EC-1 or Eca-109 cells’ groups (Figure 5D), supporting that down regulation of PTTG1 inhibited the activation of HH-GLI1 signaling pathway. These results indicated that PTTG1 promoted the occurrence of EMT in ESCC via activation of GLI1 *in vivo*.

### GLI1 up regulation partially rescued the occurrence of EMT in ESCC cells inhibited by down regulation of PTTG1

**Figure 6:**
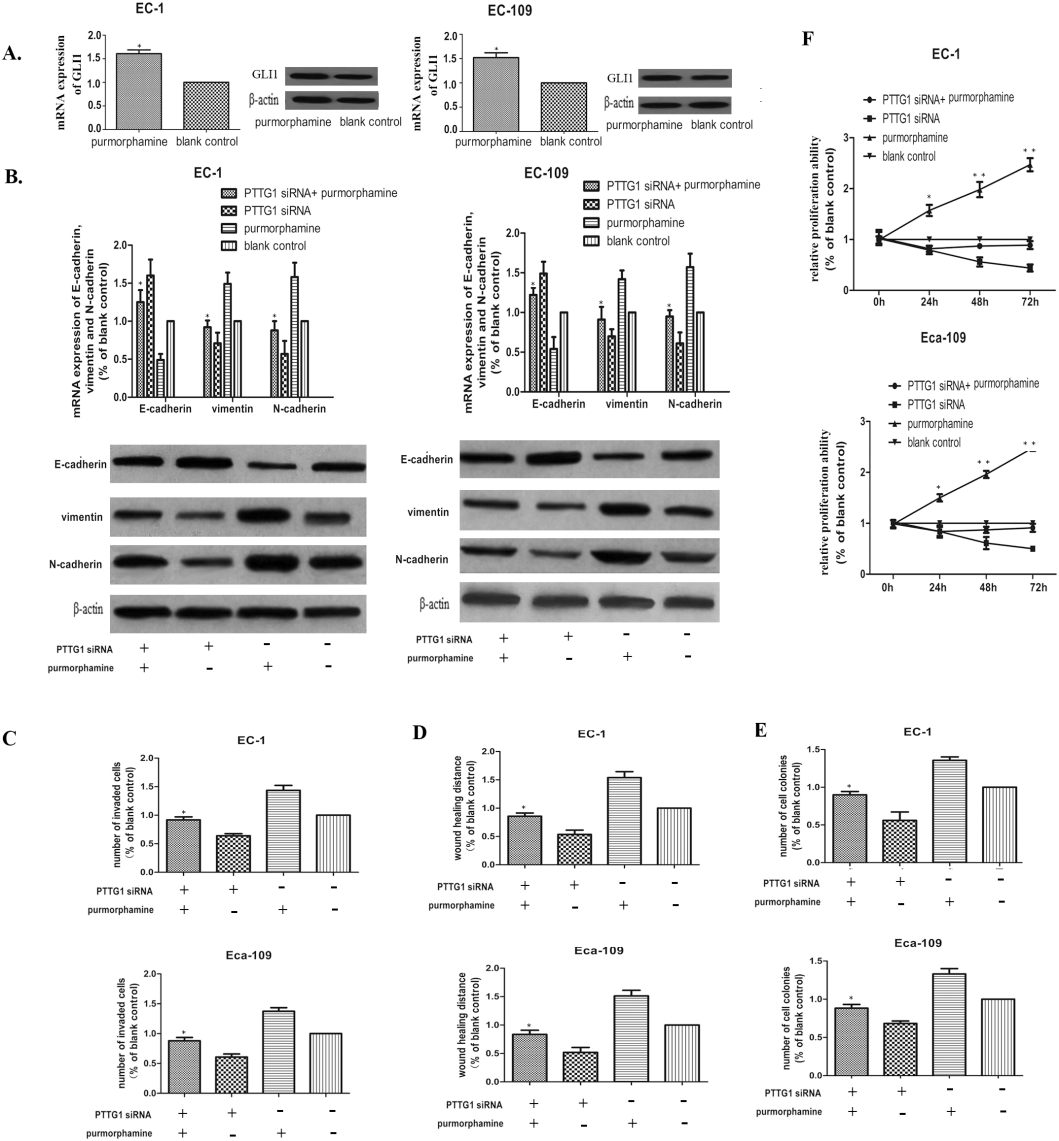
Up regulation of GLI1 partially rescued the EMT inhibited by PTTG1 siRNA **(A)** Real time RT-PCR and western blot showed that the expression of GLI1 could be promoted significantly in EC-1 and Eca-109 cells after treated with 2μg/mL purmorphamine for 48h. (^*^p<0.01 purmorphamine vs vacant cells). **(B)** Real time RT-PCR and western blot demonstrated that up regulation of GLI1 partially rescued the EMT inhibited by PTTG1 siRNA by decreasing the mRNA and protein expression of E-cadherin, while promoting the mRNA and protein expressin of vimentin and N-cadherin. (^*^p<0.05 purmorphamine vs purmorphamine+PTTG1 siRNA). **(C)** Cell invasion assay showed that up regulation of GLI1 promoted to some extent the invasion ability inhibited by PTTG1 siRNA(^*^p<0.05 purmorphamine vs purmorphamine+PTTG1 siRNA). **(D)** The results of the wound healing assay also showed that up regulation of GLI1 recovered the wound healing ability of EC-1 and Eca-109 cells partly(^*^p<0.05 purmorphamine vs purmorphamine+PTTG1 siRNA). **(E)** Colony formation assay demonstrated that to an extent, up regulation of GLI1 increased the colony formation restrained by PTTG1 siRNA(^*^p<0.05 purmorphamine vs purmorphamine+PTTG1 siRNA). **(F)** The proliferation ability of EC-1 and Eca-109 also could be rescue by up regulation of GLI1(^*^p<0.05 purmorphamine vs purmorphamine+PTTG1 siRNA).

As we have described before, purmorphamine can specifically activate the HH-GLI1 signaling pathway by up regulating the expression of GLI1 and this drug is not mitogenic or toxic to EC-1 and Eca-109 cells. Here, we validated again that the GLI1 mRNA and protein expression were all up regulated to some extent in EC-1 and Eca-109 cells treated with 2 μmol/L purmorphamine compared to blank cells (Figure 6A).

In order to comprehend the role of GLI1 in PTTG1 mediated EMT further, rescue experiment was performed by treating EC-1 and Eca-109 cells with PTTG1 siRNA and 2 μmol/L purmorphamine together for 48h, EC-1 and Eca-109 cells treated with 2 μmol/L purmorphamine, EC-1 and Eca-109 cells treated with PTTG1 siRNA and vacant EC-1 and Eca-109 cells were used as blank control. As shown in Figure 6B, up regulation of GLI1 could rescue the induction of EMT inhibited by down regulation of PTTG1 to a certain extent. The high expression of E-cadherin and low expression of vimentin and N-cadherin could be partially reversed in EC-1 and Eca-109 cells transfected with PTTG1 siRNA and 2 μmol/L purmorphamine together compared with those treated by PTTG1 siRNA separately. While the morphology of EC-1 and Eca-109 had not changed greatly. These results showed that PTTG1 promoted the occurrence of EMT partly depending on the activation of GLI1.

Furthermore, overexpression of GLI1 also promoted the metastasis, invasion and proliferation ability of EC-1 and Eca-109 cells, we used migration and invasion assays and then performed colony formation assay with these cells. We found that up regulation of GLI1 promoted invasion and migration of EC-1 and Eca-109 cells treated by PTTG1 siRNA and 2 μmol/L purmorphamine simultaneously compared with those treated by PTTG1 siRNA separately (Figure 6C, 6D). In addition, EC-1 and Eca-109 cells proliferation and colony formation ability were also reversed to a certain extent (Figure 6E, 6F). These data implicated that PTTG1 mediated EMT promoted the development of ESCC cells via activating GLI1.

## DISCUSSION

Recent studies have shown that PTTG1 could participate in cancer invasion and metastasis via promoting EMT [11–12]. However its role in ESCC had not been reported until now. In this study, we identified that PTTG1 acted as a promoter in inducing EMT and cancer metastasis in ESCC via activating GLI1, an important factor of the HH-GLI signaling pathway.

Here, we found that PTTG1 was overexpressed in ESCC tissues and ESCC cell lines. Moreover, the overexpression of PTTG1 was positively correlated with lymph node metastasis in ESCC tissues compared with those in adjacent esophageal mucosa. And the expression level of PTTG1 was also related with the invasion and metastasis ability of ESCC cells. EC-1 and Eca-109 cells with higher invasion and metastasis ability showed higher PTTG1 expression than EC9706 cells with lower invasion and metastasis ability and immortalized human esophageal epithelial cell line SHEE. In addition, down regulation of PTTG1 resulted in reduced cell invasion, metastasis, colony formation and proliferation in EC-1 and Eca-109 cells *in vitro*.

EMT is a major step during tumor invasion and metastasis by changing tumor cells from epithelial to mesenchymal phenotype. Some epithelial surface markers, such as E-cadherin are replaced by mesenchymal markers such as vimentin or N-cadherin [24–27]. Our studies demonstrated that inhibition of PTTG1 was also critical for the restrain of EMT. Down regulation of PTTG1 altered the expression of E-cadherin, vimentin or N-cadherin. Furthermore, PTTG1 deficient mice demonstrated suppression of tumor growth and EMT. Our results suggested that PTTG1 was a promoter in EMT in ESCC and PTTG1 might be an attractive target for cancer metastasis. PTTG1 had been reported to play an important role in EMT and here we also confirmed that PTTG1 induced EMT in ESCC cells and then promoted cancer metastasis. But how PTTG1 induce the occurrence of EMT haven't elucidated clearly to date.

In recent years, HH-GLI1 signaling pathway has been implicated as an important way in cancer progression and metastasis [16–18, 28]. GLI1 is the nuclear mediator of the Hedgehog pathway that regulates genes essential for various stages of tumor development and progression [19–20, 29]. In this study, we found that GLI1 was also overexpressed in ESCC tissues and cells, furthermore, the overexpression of GLI1 was also positively correlated with the invasion and metastasis ability of ESCC. Here, we demonstrated that with the down regulation of PTTG1, the mRNA and protein expression of GLI1 also decreased greatly. Additionally, we presented evidence that PTTG1 activated GLI1 via binding to its promoter. Our previous studies showed that high-expression of GLI1 dampened expression of E-cadherin and enhanced the expression of vimentin, and it also improved the expression of Snail, indicative of its role in EMT occurrence. So we thought PTTG1 could participate in EMT in ESCC via activating GLI1. In our study, we found that overexpression of GLI1 partly reversed the EMT and invasion and metastasis ability of ESCC cells blocked by down regulation of PTTG1. These results confirmed our conclusion that PTTG1 could induce EMT in ESCC via activation of GLI1.

So our study demonstrated that PTTG1 and GLI1 were all up regulated in ESCC tissue and ESCC cell lines. Furthermore, we also showed that PTTG1 promoted EMT and cancer metastasis in ESCC cell lines at least via the activation of GLI1. Based on these findings, we proposed that PTTG1 had the potential to be served as a predictive marker and a therapeutic target for ESCC.

## MATERIALS AND METHODS

### Patients and tissue samples

50 samples of formalin-fixed paraffin-embedded ESCC tissues and the corresponding normal esophageal mucosa from the First Affiliated Hospital of Zhengzhou University were chosen for our study, and informed consents were all obtained from these patients. None of these patients were treated with radiotherapy or chemotherapy before surgery. There were 34 cases with lymph node metastasis. The pathological features of patients with ESCC was shown in Supplementary Table 1.

### Immunohistochemistry

Immunohistochemistry was performed to check the PTTG1 and GLI1 protein expression in 50 human ESCC tissues and adjacent normal esophageal mucosa. Briefly, paraffin embedded specimens were baked at 60°C for 1h, then were put into xylenes and different concentration of alcohol to deparaffiniz and rehydrate. Antigenic retrieval was done by putting the slides into antigenic retrieval buffer and autoclaving for 3 minutes. Next, the slides was treated by hydrogen peroxide for 10 minutes and followed by covered with bovine serum albumin to block the nonspecific binding. Then the slides were incubated with anti-PTTG1, and anti-GLI1 rabbit antibody or anti-E-cadheirn, anti-vimentin, anti-N-cadherin *in vivo* study overnight 4°C. After washing with TBST, the slides were again incubated with anti-rabbit antibody at room temperature for 40 minutes. At last, the slides were treated by incubating with DAB, counterstained by hematoxylin, dehydrated and counted by two pathologists separately. The scores of the immunostaining on slides were multiple intensity of staining and ratio of positively stained cancer cells. Scores equal to or greater than 6 were considered as high expression.

### Cell lines, cell culture

ESCC cell lines: EC-1, EC9706 and ECa109 and immortalized human esophageal epithelial cell line SHEE were all preserved in our laboratory in the Department of Oncology, the First Affiliated Hospital of Zhengzhou University. Cell lines were cultured in RPMI-1640 medium with 10% fetal bovine serum (FBS) in a humidified atmosphere with 5% CO_2_, 37°C. All cells were enabled to attach overnight prior to transfection.

### Cell treatment and transfection

siRNA targeting PTGG1 was chemically synthesized by Shanghai Jima Corporation. For transfection, cells were cultured to 70% confluence and transfected with 100 nM PTTG1 siRNA and 100 nM scrambled siRNA (negative control) using lipofectamine 2000 according to the manufacturer's protocols, vacant cells without transfection were used as blank control. After 48h, all cells were harvested for following experiments. HH-GLI1 signaling pathway agonist purmorphamine was purchased from TESTMART Co. For rescue assay, 2 μmol/L purmorphamine and PTTG1 siRNA were used at the same time to EC-1 and Eca-109 cells, EC-1 and Eca-109 cells treated by 2 μmol/L purmorphamine, EC-1 and Eca-109 cells transfected by PTTG1 siRNA respectively or vacant EC-1 and Eca-109 cells were used as control. 48h after transfection, cells were also harvested for the additional experiments.

### Real-time RT-PCR

Total RNA was extracted by using TRIzol reagent according to the manufacture's recommendation. cDNA was generated from 1 μg total RNA by using the AMV first strand cDNA synthesis kit according to the manufacturer's instructions. cDNA product was then used for real–time PCR amplification by using latinum Taq DNA polymerase with the following primers sequences. PTTG1 forward primer: CTCGGACTGCTAACTGGACC, reverse primer: AAACAGCGGAACAGTCACGG; GLI1 forward primer: CTCCTCCCGAAGGACAGGTA, reverse primer: CATCTTGTGCATGGGACTGC; E-cadherin forward primer: CTCAAAGCCCAGAATCCCCA, reverse primer: CGGTTTTCTGTGCACACCTG; vimentin forward primer: TCCGCACATTCGAGCAAAGA, reverse primer: ATTCAAGTCTCAGCGGGCTC; N-cadherin forward primer: GCCAGAAAACTCCAGGGGAC, reverse primer: TGGCCCAGTTACACGTATCC. Relative expression was determined by the 2 (-ΔΔCt) method and real-time PCR was done in triplicate.

### Western blot

Whole cells were harvested and lysed in RIPA buffer for protein extraction. Total protein concentration was determined by using BCA kit. Subsequently, 50 μg total protein was separated by SDS-PAGE and transferred to PVDF membranes by electro method. After washing for 4 times by TBST, PVDF membranes were submerged in 5% fat-free milk for 2 h to block nonspecific binding and then incubated with PTTG1, GLI1, E-cadherin, vimentin, N-cadherin or β-actin antibody overnight at 4°C. The PVDF membranes were incubated with horseradish peroxidase-conjugated anti-goat or anti-rabbit secondary antibody again after completely washing for 1h. After exposure, PTTG1, GLI1, E-cadherin, vimentin, N-cadherin or β-actin protein expression could be analyzed by imaging analysis system. The Western blot assay was repeated for three times.

### Cell invasion assay

Transwell membrane coated with matrigel matrix was used for the invasion assay as described previously [30]. Briefly, ESCC cells (1 × 10^5^ cells/well) in RPMI-1640 medium with 1% FBS were added to the upper side of the chambers, PRMI-1640 medium with 10% FBS were loaded to the lower side of the chambers. Then the cells in chambers were put in the incubator and cultured in a humidified atmosphere with 5% CO_2_, 37°C for 48h. Cells on the upper side were moved away by cotton swabs, while the cells on the lower side after fixed and stained were calculated under microscopy at 200 × magnification. ESCC cells that invaded through each membrane were counted in five randomly selected fields. All of the experiments were done in triplicate.

### Wound healing assay

ESCC cells were seeded into 24-well plates at 70% confluence. 1mm width wound was made with a 200-μl pipette on the cell monolayer. After three times washing with PBS, the ESCC cells were cultured again in atmosphere with 5% CO_2_, 37°C for 48h, RPMI-1640 Medium was exchanged daily. Then cell migrating distances were measured under microscope at 100 × magnification by Image-Pro Plus 6.0 software.

### Colony formation assay

Cell proliferation assays were done as previously described after transfection or treatment for 48h. ESCC cells were resuspended in 0.3% agar, seeded as single cells into 10-cm culture dishes containing a layer of 0.8% agar at a density of 5000 cells per dish and then cultured in atmosphere with 5% CO_2_, 37°C for 14 days for colony formation. Then the colonies in each dish were counted and photographed. The experiments were repeated for three times.

### Cell growth assay

Cell proliferation was done by using the 3-(4.5-methylthiozol-2yl)-2.5-diphenyltetrazolium bromide (MTT) assay. Briefly, ESCCE cells (1 × 103 cells/well) were placed into 96-well plates and cultured for 24h, 48h and 72h separately after treatment or transfection. At each time point 10 μl MTT were putted into each well and the ESCC cells were cultured for another 4h. Then 200 μl DMSO were used to replace the medium in each well. The absorbance at 450 nm was measured and cell growth curves were done based on the absorbance at 450 nm. The MTT experiment was done for three times.

### Immunofluorescence assay

Cells were prepared on 0.1% gelatin-coated glass cover-slips and adherent cells were fixed in 4% paraformaldehyde for 15 min, blocked with 10% goat serum containing 0.5% Triton X-100 in PBS for 30 min, and then incubated with mouse monoclonal anti-E-cadherin antibody, anti-vimentin antibody and anti-N-cadherin antibody overnight at 4°C. After three rinses with PBS, the samples were incubated with Cy3-conjugated goat anti-mouse secondary antibody (1:100) for 1 h at room temperature in the dark. The slides were then washed in PBS and stained with 300 nM diamidinophenylindole for 10 min followed by three rinses with PBS. After mounting with fluorescent mounting media, the slides were observed under a fluorescence microscope.

### Xenograft experiments

Xenograft experiment was performed as described previously [31]. The use of SCID mice was approved by the committee of the First Affiliated Hospital of Zhengzhou University and the xenograft experiment was done in strict compliance with the regulations. Briefly, EC-1 and Eca-109 cells were injected subcutaneously into the right flank of each mouse with a concentration of 1.0^*^10^7^. After the tumor grew to 5 mm in diameter, the mice were divided randomly into three groups with 5 mice in each group. One group was treated by injection of PTTG1 siRNA (using EntransterTM-*in vivo*) one by scramble siRNA every 6 days and the last one remained untreated. The tumor-bearing mice were sacrificed 35 d after inoculation and the tumors were taken and weighed. Real time PCR and immunohistochemistry were performed to examine GLI1, EMT markers in xenograft tumour tissues.

### Chromatin immunoprecipitation(ChIP)

About 3×10^6^ EC-1 or Eca-109 cells were cross-linked and lysed using ChIP kit. Then cross-linked DNA-protein complexes were sheared to an average size of 300 to 700 bp fragments. The sheared mixture was incubated overnight with 4 μg PTTG1 antibody at 4°C. Histone H3 antibody and IgG were used as positive and negative control separately. DNA recovery was performed according to the protocol and the recovered DNA was examined by PCR using the final DNA precipitate. Sequences of primers for GLI1 promoter (targeting -841 to -665 region) were as following: forward primer: ATATGTCCAGCCCCAACTCC, reverse primer: CCAACGGCAGTCAGTTTCAT. The promoter region of GLI1 not containing the putative binding site of PTTG1 was also synthesized as negative primers.

### Statistical analysis

The data were calculated by SPSS 13.0 software for analysis. Student's t test or analysis of variance (ANOVA) was used to determine the statistical significance. p<0.05 was considered statistically significant. Data was expressed as mean ± SEM.

## SUPPLEMENTARY MATERIALS TABLE


