# Supplementary material for: PTTG1 cooperated with GLI1 leads to epithelial-mesenchymal transition in esophageal squamous cell cancer
Source: Oncotarget. 2017 Sep 27;8(54):92388–400. doi: 10.18632/oncotarget.21343 (PMC5696190; doi:10.18632/oncotarget.21343)
Supplement: Supplementary file 1 [file oncotarget-08-92388-s001.pdf]

PTTG1 cooperated with GLI1 leads to epithelial-mesenchymal transition in esophageal squamous cell cancer

SUPPLEMENTARY MATERIALS

Supplementary Table 1: Pathological information of patients with ESCC

Factors	Numbers of patients
Numbers of patients	50
Gender	
male	28
female	22
Ages(years)	
<60	20
≥60	30
Histological grades	
I	15
II	20
III	15
Invasion depth	
Superficial	21
Deep	29
Lymph node metastasis	
Yes	34
No	16
